# Water extract of the fruits of *Alpinia oxyphylla* inhibits osteoclast differentiation and bone loss

**DOI:** 10.1186/1472-6882-14-352

**Published:** 2014-09-23

**Authors:** Hyunil Ha, Ki-Shuk Shim, Taesoo Kim, Chung-Jo Lee, Ji Hyung Park, Han Sung Kim, Jin Yeul Ma

**Affiliations:** KM-Based Herbal Drug Development Group, Korea Institute of Oriental Medicine, Daejeon, 305-811 Republic of Korea; Department of Biomedical Engineering, Institute of Medical Engineering and Yonsei-Fraunhofer Medical Device Lab, Yonsei University, Wonju, Gangwon 220-710 Republic of Korea

**Keywords:** Osteoclast, *Alpinia oxyphylla*, RANKL, Bone loss

## Abstract

**Background:**

Excessive bone resorption by osteoclasts causes pathological bone destruction, seen in various bone diseases. There is accumulating evidence that certain herbal extracts have beneficial effects on bone metabolism. The fruits of *Alpinia oxyphylla* has been traditionally used for the treatment of diarrhea and enuresis. In this study, we investigated the effects of water extract of the fruits of *Alpinia oxyphylla* (WEAO) on osteoclast differentiation and osteoclast-mediated bone destruction.

**Methods:**

For osteoclast differentiation assay, mouse bone marrow-derived macrophages (BMMs) were cultured in the presence of RANKL and M-CSF. RANKL signaling pathways and gene expression of transcription factors regulating osteoclast differentiation were investigated by real-time PCR and Western blotting. A constitutively active form of NFATc1 was retrovirally transduced into BMMs. Bone resorbing activity of mature osteoclast was examined on a plate coated with an inorganic crystalline calcium phosphate. The *in vivo* effect against bone destruction was assessed in a murine model of RANKL-induced osteoporosis by micro-computed tomography and bone metabolism marker analyses.

**Results:**

WEAO dose-dependently inhibited RANKL-induced osteoclast differentiation from BMMs by targeting the early stages of osteoclast differentiation. WEAO inhibited RANKL-induced expression of NFATc1, the master regulator of osteoclast differentiation. Overexpression of a constitutively active form of NFATc1 blunted the inhibitory effect of WEAO on osteoclast differentiation, suggesting that NFATc1 is a critical target of the inhibitory action of WEAO. WEAO inhibited RANKL-induced expression of c-Fos, an upstream activator of NFATc1, by suppressing the classical NF-κB signaling pathway. WEAO also inhibited RANKL-induced down-regulation of Id2 and MafB, negative regulators of NFATc1. WEAO does not directly affect bone resorbing activity of mature osteoclasts. In accordance with the *in vitro* results, WEAO attenuated RANKL-induced bone destruction in mice by inhibiting osteoclast differentiation.

**Conclusions:**

This study demonstrates that WEAO exhibits a protective effect against bone loss by inhibiting RANKL-induced osteoclast differentiation. These findings suggest that WEAO might be useful for the prevention and treatment of bone diseases associated with excessive bone resorption.

## Background

Bone is a dynamic organ that undergoes continuous remodeling through osteoclast-mediated bone resorption and osteoblast-mediated bone formation. The bone remodeling process is highly controlled by various factors and is necessary to repair damaged bone and to maintain mineral homeostasis [1, 2].

Osteoclasts are unique, multinucleated giant cells that form through fusion of monocyte/macrophage lineage precursor cells. Macrophage colony-stimulating factor (M-CSF) and receptor activator of NF-κB ligand (RANKL) are critical cytokines required for osteoclast differentiation [3–5]. M-CSF supports the survival and proliferation of osteoclast precursors [6]. In addition, M-CSF stimulates the expression of RANK, the receptor for RANKL, in osteoclast precursors [5]. The binding of RANKL to RANK receptor on osteoclast precursors activate down downstream signaling pathways including mitogen-activated protein kinases (MAPKs) and NF-κB by recruiting adaptor molecules such as tumor necrosis factor receptor-associated factor 6, which regulate transcription factors involved in osteoclast differentiation [7–10]. Accumulating evidence suggests that nuclear factor of activated T cells cytoplasmic 1 (NFATc1) integrates RANKL signaling for terminal differentiation of osteoclasts [1]. Conditional deletion of NFATc1 in the myeloid cell lineage results in osteoclast-poor osteopetrosis, and NFATc1-deficient osteoclast precursors fails to differentiate into osteoclasts *in vitro* [11]. In addition, overexpression of NFATc1 can induce the differentiation of osteoclast precursors into mature osteoclasts even in the absence of RANKL [12]. Thus, NFATc1 is considered the master regulator of osteoclast differentiation. RANKL stimulates the expression and activation of NFATc1 in osteoclast precursors by regulating other transcription factors. It has been shown that RANKL-induced NFATc1 expression depends on NF-κB and c-Fos, which are activated by RANKL [9, 12]. It was also reported that RANKL suppresses the expression of transcriptional repressors such as inhibitors of differentiation/DNA binding (Ids), v-maf musculoaponeurotic fibrosarcoma oncogene family protein B (MafB), and interferon regulatory factor 8 that inhibit NFATc1 expression and osteoclast differentiation [13].

The RANKL/RANK pathway is not only crucial for normal bone remodeling but also primarily responsible for pathological bone destruction in various bone diseases such as postmenopausal osteoporosis, Paget’s disease, rheumatoid arthritis, and lytic bone metastasis. Thus, the RANKL/RANK pathway is considered to be an attractive therapeutic target for bone destructive diseases [2]. There is a growing interest in herbal resources including common vegetables and fruits as a promising approach for the prevention and treatment of bone-related disorders such as osteoporosis [14, 15]. We evaluated the inhibitory effects of various herbal extracts on RANKL-induced osteoclast differentiation from its precursor cells as a screen for potential herbal candidates with bone protective properties. Water extract of the fruits of *Alpinia oxyphylla* (WEAO) was found to have a relatively strong inhibitory activity against osteoclast differentiation. The fruits of *A. oxyphylla,* called Yizhi, Yakuchi, and Ikji in Chinese, Japanese, and Korean, respectively, has been traditionally used for the treatment of diarrhea and enuresis [16]. Pharmacological studies have shown that the fruits of *A. oxyphylla* possess a wide range of biological activities, including anti-diarrheal [17], anti-tumor [18], anti-anaphylactic [19], anti-ulcer [20], and neuroprotective [21, 22] activities. However, the effect of the fruits of *A. oxyphylla* on bone metabolism has not been studied. Here, we have investigated the anti-osteoclastogenic effect and action mechanism of WEAO.

## Methods

### Reagents and antibodies

Fetal bovine serum (FBS), α-modified minimal essential medium (α-MEM), and penicillin/streptomycin were purchased from Gibco BRL Life Technologies (Grand Island, NY, USA). Recombinant M-CSF and RANKL were obtained as described previously [23]. 1α,25-dihydroxyvitamin D3, prostaglandin E2, and chrysin were obtained from Sigma-Aldrich (St. Louis, MO, USA). Tectochrysin and nootkatone were obtained from ChemFaces (Wuhan, China). Antibodies against phospho-JNK1/2 (Thr183/Tyr185), JNK, phospho-p38 (Thr180/Tyr182), p38, phospho-IκBα (Ser32), and IκBα were obtained from Cell singling Technology (Danvers, MA, USA). Antibodies against NFATc1 and c-Fos were purchased from Santa Cruz biotechnology (Santa Cruz, CA, USA).

### Preparation of WEAO

Air-dried fruits of *A. oxyphylla* were obtained from Yeongcheon Oriental Herbal Market (Yeongcheon, Korea) and authenticated by an expert botanist, emeritus Prof. Ki-Hwan Bae (Collage of Pharmacy, Chungnam National University). A voucher specimen was deposited at the herbarium of the KM-Based Herbal Drug Development Group, Korea Institute of Oriental Medicine. The materials were extracted by boiling in distilled water (1:10, w/v) for 3 h. The water extract was filtered with standard sieves (106 μm; Restsch, Haan, Germany) and then lyophilized. The lyophilized powder (yield: 5.52% of dried fruits) was re-suspended in distilled water and centrifuged at 10,000 g for 5 min to prepare WEAO. For *in vitro* experiments, WEAO was filtered through a 0.2 μm filter.

### High performance liquid chromatography (HPLC) analysis

HPLC analysis of WEAO was conducted using a Hitachi LaChrom Elite HPLC system (Hitachi High Technologies Corp., Tokyo, Japan). The chromatographic separation was carried out using a Kinetex C_8_ column (4.6 mm × 100 mm, 2.6 μm, 40 °C). The mobile phase was 0.1% trifluoroacetic acid in deionized water (A) and acetonitrile (B) with a step gradient elution (0–3 min, 5%–5% B; 3–63 min, 5%–70% B; 63–73 min, 70%–70% B). A mixture of standard compounds (Chrysin, Tectochrysin, Nootkatone; each 200 μg/mL) and WEAO (100 mg/mL) were dissolved in methanol and filtered through a 0.2 μm syringe filter prior to injection for HPLC analysis.

### Osteoclast differentiation assay

Bone marrow cells were collected by flushing the femurs and tibias from 6- to 7-week-old male ICR mice with α-MEM. After removing red blood cells, BMMs were prepared from bone marrow cells with M-CSF (60 ng/mL) in α-MEM complete medium containing 10% FBS and 1% penicillin/streptomycin as described previously [24]. For osteoclast differentiation, BMMs (1 × 10^4^ cells/well in a 96-well plate) were cultured in α-MEM complete medium supplemented with M-CSF (60 ng/mL) and RANKL (100 ng/mL) for 4 days. To investigate the effect of WEAO on osteoclast differentiation, WEAO was added to the cultures simultaneously with RANKL at day 0 or after 1, 2, or 3 days. Cells were replenished with fresh medium and treatments on the third day of culture. To assess osteoclast differentiation, cells were stained for tartrate-resistant acid phosphatase (TRAP) activity as described below. TRAP-positive multinucleated cells containing more than three nuclei and larger than 100 μm in diameter were counted as osteoclasts.

### TRAP assay

TRAP staining was performed using TRAP buffer (50 mM sodium tartrate and 0.12 M sodium acetate, pH 5.2) with naphthol AS-MX phosphate and fast red violet LB salt (Sigma-Aldrich) as described previously [23]. Serum TRAP isoenzyme 5b (TRAP 5b) activity was determined using the fluorogenic substrate, naphthol AS-BI phosphate (Sigma-Aldrich). In brief, 20 μL of serum was incubated with 100 μL of TRAP 5b reaction buffer (2.5 mM naphthol AS-BI phosphate, 50 mM sodium tartrate and 0.12 M sodium acetate, 2% NP-40, and 1% ethylene glycol monomethyl ether, pH 6.1) for 30 min at 37°C, and the reaction was stopped by adding 120 μL of 0.1 M NaOH. Fluorescence was measured at an excitation wavelength of 405 nm and an emission wavelength of 520 nm.

### Cell viability assay

BMMs (1 × 10^4^ cells/well in a 96-well plate) were cultured with or without WEAO (10–160 μg/mL) in the presence of M-CSF (60 ng/mL) for 2 days, and the viability of BMMs was evaluated with Cell Counting Kit-8 assay (Dojindo Molecular Technologies Inc., Rockville, MD, USA) according to the manufacturer’s protocol.

### Animals and RANKL-induced osteoporosis model

All animal experiments were carried out according to the protocols (permission number: 12–090) approved by the Institutional Animal Care and Use Committee Guidelines of Korea Institute of Oriental Medicine. 5-week-old male ICR mice were purchased from Orient Bio Inc. (Seoul, Korea) and housed under constant environmental conditions (22 ± 1°C, 55 ± 10% humidity, and 12 h light/dark cycle) with free access to a standard animal diet and water. 7-week-old mice were orally administered with vehicle (distilled water) or WEAO (0.25 g/kg of body weight) twice daily for consecutive 5 days, and RANKL (1 mg/kg of body weight) or PBS was intraperitoneally injected on days 3 and 4. On day 6, fasting blood samples and the right femurs were collected.

### Measurement of serum markers of bone resorption and bone formation

Serum levels of C-terminal cross-linked telopeptide of type I collagen (CTX) and osteocalcin were measured using a RatLaps EIA kit (Immunodiagnostic Systems Inc., Fountain Hills, AZ, USA) and a mouse osteocalcin EIA kit (Biomedical Technologies Inc., Stoughton, MA, USA), respectively.

### Micro-computed tomography (micro-CT) analysis

Micro-CT scanning of the distal femur was carryout out on SkyScan 1076 CT scanner system (SkyScan N.V., Kontich, Belgium). The scanned images were reconstructed and analyzed using SkyScan CT Analyzer (Skyscan). The structural parameters including trabecular bone volume per tissue volume, thickness, separation, and number were measured in the distal femoral metaphysis between 0.54 and 1.46 mm distal to the growth plate.

### Bone resorption assay

Primary pre-osteoblasts were obtained from calvariae of newborn ICR mice (Orient Bio Inc.) by a sequential enzymatic digestion method described previously [25]. To obtain mature osteoclasts, bone marrow cells (1.5 × 10^7^ cells) and pre-osteoblasts (1.5 × 10^6^ cells) were cocultured in the presence of 1α,25-dihydroxyvitamin D3 (10 nM) and prostaglandin E2 (100 nM) in a 10-cm dish coated with collagen gel (Cellmatrix type I-A, Nitta Gelatin Inc., Osaka, Japan) for 6 days. The plates were treated with collagenase (Sigma-Aldrich), and mature osteoclasts were placed on an Osteo Assay Surface plate (Corning Inc., Corning, NY, USA), allowed to settle for 2 h, and then cultured with or without WEAO. After 24 h of culture, cells were stained for TRAP activity. Resorption pits by osteoclasts were photographed and analyzed by using ImageJ software, after removing cells with sodium hypochlorite.

### Quantitative real-time polymerase chain reaction (qPCR) analysis

Total RNA was extracted with RNA-spin total RNA Extraction Kit (Bioneer, Daejeon, Korea), and fist-strand cDNA was synthesized from 1 μg of total RNA with AccuPower RT-PreMix (Bioneer). SYBR green-based qPCR was performed on the Applied Biosystems 7500 Real-Time PCR System (Foster City, CA, USA) with cDNA diluted to 1:5, 10 pmol of primers, and AccuPower GreenStar qPCR Master Mix (Bioneer). The following mouse-specific primer sets were used: c-Fos, 5′-CGGGTTTCAACGCCGACTAC-3′ (forward) and 5′-AAAGTTGGCACTAGAGACGGACAGA-3′ (reverse); NFATc1, 5′-CCGTTGCTTCCAGAAAATAACA-3′ (forward) and 5′-TGTGGGATGTGAACTCGGAA-3′ (reverse); Id2, 5′-CGGTGAGGTCCGTTAGGAAAA-3′ (forward) and 5′-CATGTTGTAGAGCAGACTCATCG-3′ (reverse); MafB, 5′-AGTGTGGAGGACCGCTTCTCTG-3′ (forward) and 5′-CTGGACGCGTTTATACCTGC-3′ (reverse); hypoxanthine phosphoribosyltransferase, 5′-CCTAAGATGAGCGCAAGTTGAA-3′ (forward) and 5′-CCACAGGACTAGAACACCTGCTAA-3′ (reverse). All reactions were run in triplicate, and data were analyzed using the 2^-ΔΔCT^ method. Hypoxanthine phosphoribosyltransferase was used as an internal control.

### Western blot analysis

Cells were washed with ice-cold PBS and lysed with RIPA buffer (Millipore, Temecula, CA, USA) supplemented with protease-inhibitor and phosphatase-inhibitor cocktail tablets (Roche Applied Science, Indianapolis, IL, USA) on ice for 30 min. The cell extracts were centrifuged at 10,000 g, 4°C for 10 min. Equal amounts of proteins (30 μg) were boiled in sodium dodecyl sulfate (SDS) sample buffer for 5 min and separated by 10% SDS-polyacrylamide gel electrophoresis and transferred to a polyvinylidene fluoride membrane (GE Healthcare, Buckinghamshire, UK). After blocking with 5% nonfat dry milk, the membranes were probed with the indicated primary antibodies, followed by secondary antibodies conjugated with horseradish peroxidase. Immunoreactive bands were visualized with SuperSignal West Femto Maximum Sensitivity Substrate (Thermo Fisher Scientific Inc., Rockford, IL, USA) using Luminescent Image Analyzer LAS-4000 (Fuji Photo Film Co., Tokyo, Japan).

### Retroviral gene transduction

Retrovirus packaging and BMM infection by using retroviral vectors pMX-IRES-green fluorescent protein (GFP) and pMX-constitutively active (CA)-NFATc1-IRES-GFP were performed as described previously [24]. In brief, retrovirus packaging was performed by transient transfection of these pMX vectors into Plat-E retroviral packaging cells (Cell Biolabs, San Diego, CA, USA). BMMs were incubated with the viral supernatant from Plat-E cells together with polybrene (6 μg/mL, Sigma-Aldrich) and M-CSF (60 ng/mL) for 12 h. After removing viral supernatant, BMMs were cultured in the presence of M-CSF for 1 day and then treated as indicated.

### Statistical analysis

Values are presented as mean ± SD in *in vitro* study and mean ± SEM in *in vivo* study. Two-group comparisons were performed with Student's *t* tests, while multiple-group comparisons were performed with analysis of variance followed by Dunnett’s test. A *p*-value less than 0.05 was considered statistically significant.

## Results and discussion

### WEAO inhibits RANKL-induced osteoclast differentiation

**Figure 1:**
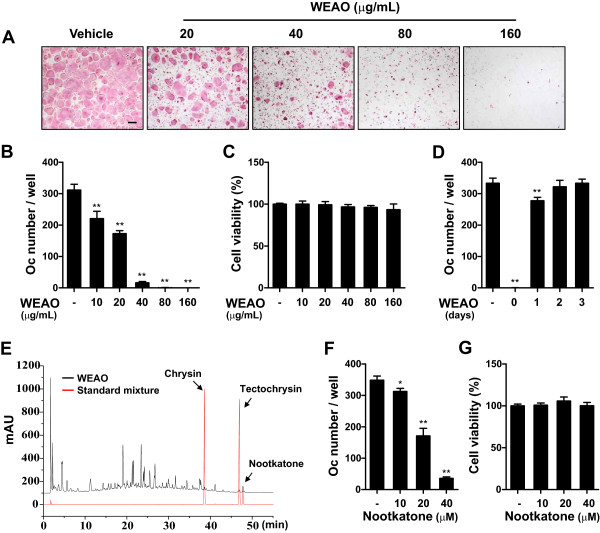
**Effect of WEAO on RANKL-induced osteoclast differentiation in BMMs. (A, B)** BMMs were cultured with vehicle (distilled water) or WEAO (10–160 μg/mL) in the presence of M-CSF (60 ng/mL) and RANKL (100 ng/mL) for 4 days. **(A)** Cells were fixed and stained for TRAP activity. Scale bar, 200 μm. **(B)** The number of osteoclasts (Oc) was counted. **(C)** The viability of BMMs was determined by Cell Counting Kit-8 assay. **(D)** BMMs were cultured in the presence of M-CSF and RANKL for 4 days, and WEAO (80 μg/mL) was added to the BMM cultures at the indicated days. The number of osteoclasts was counted on day 4. **(E)** HPLC chromatograms of WEAO and a standard mixture of chrysin, tectochrysin, and nootkatone at 203 nm. **(F)** BMMs were cultured with vehicle (dimethylsulfoxide) or nootkatone (10–40 μM) in the presence of M-CSF and RANKL for 4 days, and the number of osteoclasts was counted. **(G)** The effect of nootkatone on viability of BMMs. *p < 0.05; **p < 0.01 versus vehicle-treated control.

To determine whether WEAO affects osteoclast differentiation, we examined the effect of WEAO on RANKL-induced osteoclast differentiation from its precursor cells, BMMs. Treatment of BMMs with M-CSF and RANKL for 4 days induced TRAP-positive multinucleated osteoclasts. When WEAO was added to the BMM cultures simultaneously with RANKL, osteoclast differentiation was inhibited by WEAO in a dose-dependent manner (Figure 1A and B). WEAO did not affect the viability of BMMs at concentrations less than 160 μg/mL (Figure 1C), indicating that the inhibitory effect of WEAO on osteoclast differentiation did not result from cytotoxicity. WEAO at 80 μg/mL almost completely inhibited osteoclast differentiation, without affecting cell viability. Thus, 80 μg/mL of WEAO was used for the subsequent experiments to elucidate the mechanisms underlying its anti-osteoclastogenic effect. To determine at which stage WEAO inhibits osteoclast differentiation, WEAO was added to the BMM cultures on days 0–3. WEAO effectively inhibited RANKL-induced osteoclast differentiation when added simultaneously with RANKL (day 0). In contrast, WEAO did not affect RANKL-induced osteoclast differentiation when added on days 2 and 3 (Figure 1D). These results suggest that WEAO inhibits early cellular events for RANKL-induced osteoclastogenesis.

Previous phytochemical studies have shown that flavonoids (e.g., tectochrysin and chrysin), diarylheptanoids (e.g., yakuchinone A and yakuchinone B), and sesquiterpenes (e.g., nootkatone) are abundant in ethanol extracts of the fruits of *A. oxyphylla* [17, 26]. In accordance with this, we identified chrysin, tectochrysin, and nootkatone in 75% ethanol extract of the fruits of *A. oxyphylla* HPLC analysis, based on their HPLC retention times and UV absorption spectra (data not shown). Among these compounds, nootkatone was relatively abundant in WEAO (Figure 1E). Nootkatone has been shown to have multiple pharmacological properties including anti-inflammatory [27], anti-allergic [28], antiplatelet [29], and anti-obesity [30] activities. We found that nootkatone (10–40 μM) dose-dependently inhibits RANKL-induced osteoclast differentiation without affecting cell viability (Figure 1F and G). Although further study is needed to isolate and characterize the chemical constituents of WEAO, our results suggest that nootkatone might be one of the active constituents contributing to the inhibitory effect of WEAO on osteoclast differentiation.

### WEAO inhibits RANKL-induced NFATc1 expression in osteoclast precursors

**Figure 2:**
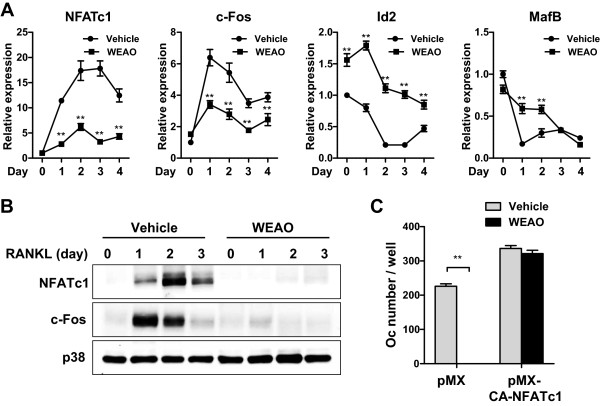
**Effect of WEAO on the gene expression of transcription factors involved in osteoclast differentiation. (A, B)** BMMs were cultured with vehicle (distilled water) or WEAO (80 μg/mL) in the presence of M-CSF (60 ng/mL). Total RNA and cell lysates were obtained following stimulation with RANKL (100 ng/mL) for the indicated times. **(A)** Relative mRNA expression levels of NFATc1, c-Fos, Id2, and MafB were determined by qPCR. **p < 0.01 versus vehicle-treated control. **(B)** Total cell lysates were subjected to Western blot analysis with antibodies against NFATc1, c-Fos, and p38. p38 was used as a loading control. **(C)** BMMs transduced with retroviruses expressing either pMX-IRES-GFP (pMX) or pMX-CA-NFATc1-IRES-GFP (pMX-CA-NFATc1) were cultured with vehicle or WEAO (80 μg/mL) in the presence of M-CSF (60 ng/mL) and RANKL (100 ng/ml) for 4 days. The number of osteoclasts (Oc) was counted. **p < 0.01.

We next investigated the effect of WEAO on the expression of transcription factors regulating osteoclast differentiation. RANKL stimulated the mRNA and protein expression of NFATc1 in BMMs, and WEAO blunted RANKL-induced NFATc1 expression (Figure 2A and B). To investigate whether the reduction of NFATc1 is responsible for the anti-osteoclastogenic action of WEAO, BMMs were transduced with retroviruses harboring control GFP or CA-NFATc1-GFP vectors. WEAO blocked RANKL-induced osteoclast differentiation in BMMs infected with control GFP, whereas the ectopic expression of CA-NFATc1 rescued the inhibitory effect of WEAO (Figure 2C). These results suggest the involvement of NFATc1 in the anti-osteoclastogenic effect of WEAO and further confirmed that the anti-osteoclastogenic effect of WEAO is not due to cytotoxicity.

The transcription factor c-Fos is also essential for osteoclastogenesis and functions as a key upstream activator of NFATc1 during osteoclastogenesis [31]. Similar to its effect on NFATc1 expression, WEAO abrogated RANKL-induced c-Fos mRNA and protein expression (Figure 2A and B). In addition to up-regulation of transcriptional activators, RANKL stimulates NFATc1 expression and osteoclastogenesis by suppression of several transcriptional repressors including Ids and MafB [13]. The expression of Id helix-loop-helix transcription factors is suppressed by RANKL during osteoclastogenesis, and Ids negatively regulate RANKL-induced osteoclastogenesis by down-regulating the expression of osteoclast-associated receptor and NFATc1 through attenuation of DNA binding ability of Mitf [32]. MafB expression is also down-regulated by RANKL, and MafB suppresses osteoclastogenesis by interfering with transcriptional activities of c-Fos, Mitf, and NFATc1 [33]. Consistent with the previous reports, RANKL markedly reduced the expression of Id2 and MafB in BMMs. WEAO increased Id2 expression and inhibited RANKL-induced down-regulation of Id2 and MafB expression (Figure 2A). It has been shown that Id2 and MafB do not affect RANKL-induced c-Fos expression [33, 34]. Thus, these results collectively suggest that WEAO inhibits RANKL-induced NFATc1 expression by suppressing the induction of its up-stream activator c-Fos as well as the down-regulation of its negative regulators, Id2 and MafB, thereby inhibiting osteoclastogenesis.

### WEAO alters RANKL-induced activation of MAPKs and NF-κB

**Figure 3:**
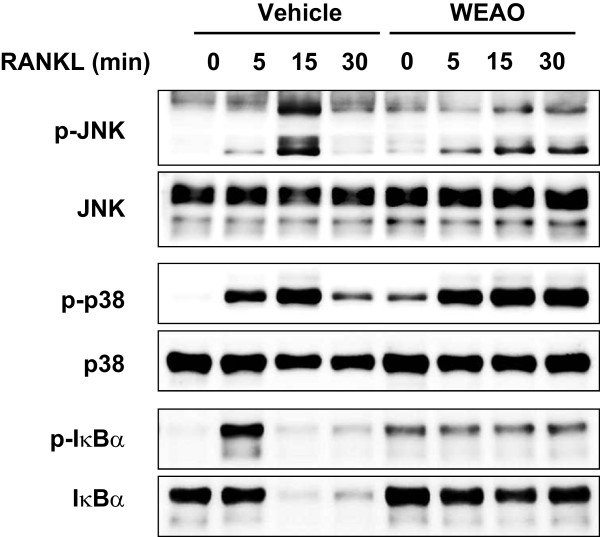
**Effect of WEAO on RANKL-induced activation of MAPKs and NF-κB in BMMs.** BMMs were pretreated with vehicle or WEAO (80 μg/mL) for 3 h and then stimulated with RANKL (100 ng/mL) for the indicated times. Total cell lysates were subjected to Western blot analysis using the indicated antibodies.

Binding of RANKL to RANK leads to the activation of MAPK and NF-κB pathways, which are implicated in osteoclast differentiation by involving c-Fos and NFATc1 induction [7–9, 35, 36]. To get more insights into the mechanisms underlying the anti-osteoclastogenic effect of WEAO, we investigated whether WEAO affects the early signaling events trigged by RANKL. Treatment of BMMs with RANKL increased the phosphorylation of JNK and p38 MAPKs within 5 min, reached the maximum at 15 min, and then decreased thereafter (Figure 3). Pretreatment of BMMs with WEAO increased the phosphorylation of JNK and p38 and prolonged RANKL-induced phosphorylation of JNK and p38. RANKL stimulated the classical NF-κB signaling pathway, determined by IκBα phosphorylation and subsequent degradation. Pretreatment of BMMs with WEAO increased IκBα phosphorylation but markedly suppressed RANKL-induced IκBα phosphorylation and degradation (Figure 3). Thus, these results suggest that WEAO inhibits RANKL-induced c-Fos induction, at least in part, by suppressing the classical NF-κB signaling pathway.

It has been reported that RANKL down-regulates MafB expression through JNK and p38 MAPK pathways but not the classical NF-κB pathway [33]. Thus, our results suggest that WEAO inhibits RANKL-mediated MafB down-regulation independently of these MAPK pathways. However, the mechanisms underlying the inhibitory effects of WEAO on RANKL-mediated down-regulation of MafB and Id2 remain to be elucidated.

### WEAO does not affect bone resorbing activity of mature osteoclasts

**Figure 4:**
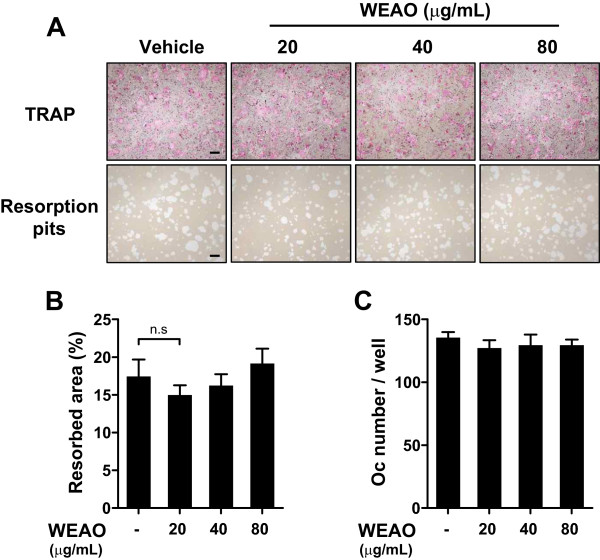
**Effect of WEAO on bone resorbing activity of mature osteoclasts.** Mature osteoclasts were seeded on a plate coated with an inorganic crystalline calcium phosphate and then cultured with vehicle or WEAO (20–80 μg/mL) for 24 h. **(A)** Cells were stained for TRAP activity, and resorption pits formed by osteoclasts were photographed. Scale bar, 200 μm. **(B)** The number of osteoclasts (Oc) was counted. n.s, not significant. **(C)** The total area of resorption pits was measured.

When attached to bone matrix, osteoclasts polarize their membrane to bone surface and secret hydrochloric acid and acidic proteases which degrade bone matrix in a sealed compartment [2]. To investigate whether WEAO affects resorbing activity of mature osteoclasts, mature osteoclasts were generated from the coculture of bone marrow cells and pre-osteoblasts and seeded on a plate coated with an inorganic crystalline calcium phosphate designed to mimic bone mineral. After 24 h of culture, numerous pits resorbed by osteoclasts were formed on the plate. WEAO did not affect osteoclast number and the resorbed area at 20–80 μg/mL (Figure 4A–C), indicating that WEAO does not directly affect bone resorbing activity of mature osteoclasts.

### WEAO inhibits RANKL-induced bone loss *in vivo*

**Figure 5:**
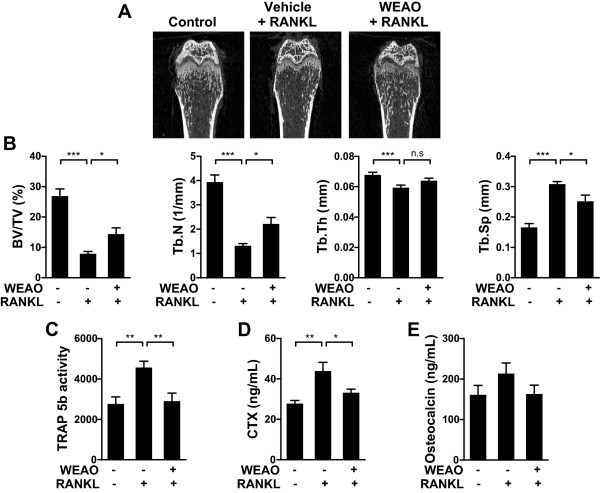
**Effect of WEAO on RANKL-induced bone destruction in mice.** Mice were orally administrated with vehicle (n = 8 per group) or WEAO (0.25 g/kg, n = 7) twice daily for 5 days, and RANKL (1 mg/kg) was injected intraperitoneally on days 3 and 4. Blood serum and right femur were collected on day 6. **(A)** Representative micro**-**CT images of the distal femurs from each group. **(B)** Quantitative micro**-**CT analysis of trabecular bone volume/tissue volume (BV/TV), trabecular number (Tb.N), trabecular thickness (Tb.Th), and trabecular separation (Tb.Sp) in the distal femoral metaphysis. **(C)** Serum TRAP 5b activity. **(D)** Serum CTX levels. **(E)** Serum osteocalcin levels. *p < 0.05; **p < 0.01; ***p < 0.001; n.s, not significant.

Increased RANKL activity is involved in bone destruction in various bone diseases [2]. Having found that WEAO inhibits RANKL-induced osteoclast differentiation *in vitro*, we next examined the *in vivo* effect of WEAO on osteoclast-mediated bone destruction using a murine model of bone loss by RANKL injection. Intraperitoneal injections of RANKL into mice rapidly induce bone loss by stimulating osteoclast differentiation and function [37]. RANKL injections caused a severe trabecular bone loss at the distal femoral metaphysis (Figure 5A). Quantitative micro-CT analysis of trabecular bone microarchitecture showed that RANKL injections led to a marked decrease in trabecular bone volume per tissue volume, trabecular thickness, and trabecular number, with an increase in trabecular separation. Oral administration of WEAO (0.25 g/kg twice daily) significantly attenuated RANKL-induced changes in trabecular bone microarchitecture, except trabecular thickness (Figure 5A and B). We next investigated the changes in serum TRAP 5b activity, CTX levels, and osteocalcin levels, which are used for markers of osteoclast numbers, bone resorption, and bone formation, respectively [38]. WEAO markedly inhibited the increases in serum TRAP 5b activity and CTX levels induced by RANKL injections (Figure 5C and D). There were no significant differences in serum osteocalcin levels among the groups (Figure 5E). Therefore, it is likely that the protective effect of WEAO against bone destruction is mainly due to suppression of bone resorption through inhibition of osteoclast differentiation. Our results clearly showed that WEAO at a dose of 0.25 g/kg twice daily attenuates RANKL-induced osteoclast differentiation and bone destruction *in vivo*. However, such beneficial effects of WEAO were not observed at a dose of 0.75 g/kg (data not shown). Therefore, further studies are needed to more thoroughly characterize the dose response effects of WEAO on bone metabolism. In addition, the beneficial effect of WEAO in bone disease states such as postmenopausal osteoporosis and lytic bone metastasis remains to be investigated.

## Conclusions

This study demonstrated that WEAO inhibits osteoclast differentiation by suppressing RANKL-induced NFATc1 expression in osteoclast precursors. The reduction of NFATc1 expression by WEAO was attributable to suppression of RANKL-induced up-regulation of its positive regulators, NF-κB and c-Fos, and down-regulation of its negative regulators, Id2 and MafB. Consistent with the *in vitro* results, oral administration of WEAO attenuated RANKL-induced bone destruction by inhibiting osteoclast differentiation. Given the role of excessive RANKL activity in pathological bone loss, our findings suggest that WEAO may be useful for the prevention and treatment of bone metabolic diseases associated with excessive osteoclastic bone resorption.

## References

[1] Negishi-Koga T, Takayanagi H (2009). Ca2 + −NFATc1 signaling is an essential axis of osteoclast differentiation. Immunol Rev.

[2] Tanaka S, Nakamura K, Takahasi N, Suda T (2005). Role of RANKL in physiological and pathological bone resorption and therapeutics targeting the RANKL-RANK signaling system. Immunol Rev.

[3] Kong YY, Yoshida H, Sarosi I, Tan HL, Timms E, Capparelli C, Morony S, Oliveira-dos-Santos AJ, Van G, Itie A, Khoo W, Wakeham A, Dunstan CR, Lacey DL, Mak TW, Boyle WJ, Penninger JM (1999). OPGL is a key regulator of osteoclastogenesis, lymphocyte development and lymph-node organogenesis. Nature.

[4] Lacey DL, Timms E, Tan HL, Kelley MJ, Dunstan CR, Burgess T, Elliott R, Colombero A, Elliott G, Scully S, Hsu H, Sullivan J, Hawkins N, Davy E, Capparelli C, Eli A, Qian YX, Kaufman S, Sarosi I, Shalhoub V, Senaldi G, Guo J, Delaney J, Boyle WJ (1998). Osteoprotegerin ligand is a cytokine that regulates osteoclast differentiation and activation. Cell.

[5] Arai F, Miyamoto T, Ohneda O, Inada T, Sudo T, Brasel K, Miyata T, Anderson DM, Suda T (1999). Commitment and differentiation of osteoclast precursor cells by the sequential expression of c-Fms and receptor activator of nuclear factor kappaB (RANK) receptors. J Exp Med.

[6] Tanaka S, Takahashi N, Udagawa N, Tamura T, Akatsu T, Stanley ER, Kurokawa T, Suda T (1993). Macrophage colony-stimulating factor is indispensable for both proliferation and differentiation of osteoclast progenitors. J Clin Invest.

[7] Ikeda F, Nishimura R, Matsubara T, Tanaka S, Inoue J, Reddy SV, Hata K, Yamashita K, Hiraga T, Watanabe T, Kukita T, Yoshioka K, Rao A, Yoneda T (2004). Critical roles of c-Jun signaling in regulation of NFAT family and RANKL-regulated osteoclast differentiation. J Clin Invest.

[8] Huang H, Chang EJ, Ryu J, Lee ZH, Lee Y, Kim HH (2006). Induction of c-Fos and NFATc1 during RANKL-stimulated osteoclast differentiation is mediated by the p38 signaling pathway. Biochem Biophys Res Commun.

[9] Yamashita T, Yao Z, Li F, Zhang Q, Badell IR, Schwarz EM, Takeshita S, Wagner EF, Noda M, Matsuo K, Xing L, Boyce BF (2007). NF-kappaB p50 and p52 regulate receptor activator of NF-kappaB ligand (RANKL) and tumor necrosis factor-induced osteoclast precursor differentiation by activating c-Fos and NFATc1. J Biol Chem.

[10] Gohda J, Akiyama T, Koga T, Takayanagi H, Tanaka S, Inoue J (2005). RANK-mediated amplification of TRAF6 signaling leads to NFATc1 induction during osteoclastogenesis. EMBO J.

[11] Aliprantis AO, Ueki Y, Sulyanto R, Park A, Sigrist KS, Sharma SM, Ostrowski MC, Olsen BR, Glimcher LH (2008). NFATc1 in mice represses osteoprotegerin during osteoclastogenesis and dissociates systemic osteopenia from inflammation in cherubism. J Clin Invest.

[12] Takayanagi H, Kim S, Koga T, Nishina H, Isshiki M, Yoshida H, Saiura A, Isobe M, Yokochi T, Inoue J, Wagner EF, Mak TW, Kodama T, Taniguchi T (2002). Induction and activation of the transcription factor NFATc1 (NFAT2) integrate RANKL signaling in terminal differentiation of osteoclasts. Dev Cell.

[13] Zhao B, Ivashkiv LB (2011). Negative regulation of osteoclastogenesis and bone resorption by cytokines and transcriptional repressors. Arthritis Res Ther.

[14] Putnam SE, Scutt AM, Bicknell K, Priestley CM, Williamson EM (2007). Natural products as alternative treatments for metabolic bone disorders and for maintenance of bone health. Phytother Res.

[15] Banu J, Varela E, Fernandes G (2012). Alternative therapies for the prevention and treatment of osteoporosis. Nutr Rev.

[16] Commission CP (2010). Pharmacopoeia of the People’s Republic of China (First Division).

[17] Zhang J, Wang S, Li Y, Xu P, Chen F, Tan Y, Duan J (2013). Anti-diarrheal constituents of Alpinia oxyphylla. Fitoterapia.

[18] Wang CZ, Yuan HH, Bao XL, Lan MB (2013). In vitro antioxidant and cytotoxic properties of ethanol extract of Alpinia oxyphylla fruits. Pharm Biol.

[19] Shin TY, Won JH, Kim HM, Kim SH (2001). Effect of Alpinia oxyphylla fruit extract on compound 48/80-induced anaphylactic reactions. Am J Chin Med.

[20] Yamahara J, Li YH, Tamai Y (1990). Anti-ulcer effect in rats of bitter cardamon constituents. Chem Pharm Bull (Tokyo).

[21] Zhang ZJ, Cheang LC, Wang MW, Li GH, Chu IK, Lin ZX, Lee SM (2012). Ethanolic extract of fructus Alpinia oxyphylla protects against 6-hydroxydopamine-induced damage of PC12 cells in vitro and dopaminergic neurons in zebrafish. Cell Mol Neurobiol.

[22] Yu X, An L, Wang Y, Zhao H, Gao C (2003). Neuroprotective effect of Alpinia oxyphylla Miq. fruits against glutamate-induced apoptosis in cortical neurons. Toxicol Lett.

[23] Ha H, An H, Shim KS, Kim T, Lee KJ, Hwang YH, Ma JY (2013). Ethanol extract of Atractylodes macrocephala protects bone loss by inhibiting osteoclast differentiation. Molecules.

[24] Lee JH, Kim HN, Yang D, Jung K, Kim HM, Kim HH, Ha H, Lee ZH (2009). Trolox prevents osteoclastogenesis by suppressing RANKL expression and signaling. J Biol Chem.

[25] Ichida F, Nishimura R, Hata K, Matsubara T, Ikeda F, Hisada K, Yatani H, Cao X, Komori T, Yamaguchi A, Yoneda T (2004). Reciprocal roles of MSX2 in regulation of osteoblast and adipocyte differentiation. J Biol Chem.

[26] Li YH, Chen F, Wang JF, Wang Y, Zhang JQ, Guo T (2013). Analysis of nine compounds from Alpinia oxyphylla fruit at different harvest time using UFLC-MS/MS and an extraction method optimized by orthogonal design. Chem Cent J.

[27] Tsoyi K, Jang HJ, Lee YS, Kim YM, Kim HJ, Seo HG, Lee JH, Kwak JH, Lee DU, Chang KC (2011). (+)-Nootkatone and (+)-valencene from rhizomes of Cyperus rotundus increase survival rates in septic mice due to heme oxygenase-1 induction. J Ethnopharmacol.

[28] Jin JH, Lee DU, Kim YS, Kim HP (2011). Anti-allergic activity of sesquiterpenes from the rhizomes of Cyperus rotundus. Arch Pharm Res.

[29] Seo EJ, Lee DU, Kwak JH, Lee SM, Kim YS, Jung YS (2011). Antiplatelet effects of Cyperus rotundus and its component (+)-nootkatone. J Ethnopharmacol.

[30] Murase T, Misawa K, Haramizu S, Minegishi Y, Hase T (2010). Nootkatone, a characteristic constituent of grapefruit, stimulates energy metabolism and prevents diet-induced obesity by activating AMPK. Am J Physiol Endocrinol Metab.

[31] Matsuo K, Galson DL, Zhao C, Peng L, Laplace C, Wang KZ, Bachler MA, Amano H, Aburatani H, Ishikawa H, Wagner EF (2004). Nuclear factor of activated T-cells (NFAT) rescues osteoclastogenesis in precursors lacking c-Fos. J Biol Chem.

[32] Lee J, Kim K, Kim JH, Jin HM, Choi HK, Lee SH, Kook H, Kim KK, Yokota Y, Lee SY, Choi Y, Kim N (2006). Id helix-loop-helix proteins negatively regulate TRANCE-mediated osteoclast differentiation. Blood.

[33] Kim K, Kim JH, Lee J, Jin HM, Kook H, Kim KK, Lee SY, Kim N (2007). MafB negatively regulates RANKL-mediated osteoclast differentiation. Blood.

[34] Oh J, Lee MS, Yeon JT, Choi SW, Kim HS, Shim H, Lee SY, Youn BS, Yokota Y, Kim JH, Kwak HB (2012). Inhibitory regulation of osteoclast differentiation by interleukin-3 via regulation of c-Fos and Id protein expression. J Cell Physiol.

[35] Lee JH, Jin H, Shim HE, Kim HN, Ha H, Lee ZH (2010). Epigallocatechin-3-gallate inhibits osteoclastogenesis by down-regulating c-Fos expression and suppressing the nuclear factor-kappaB signal. Mol Pharmacol.

[36] Iotsova V, Caamano J, Loy J, Yang Y, Lewin A, Bravo R (1997). Osteopetrosis in mice lacking NF-kappaB1 and NF-kappaB2. Nat Med.

[37] Tomimori Y, Mori K, Koide M, Nakamichi Y, Ninomiya T, Udagawa N, Yasuda H (2009). Evaluation of pharmaceuticals with a novel 50-hour animal model of bone loss. J Bone Miner Res.

[38] Naylor K, Eastell R (2012). Bone turnover markers: use in osteoporosis. Nat Rev Rheumatol.

[CR39] The pre-publication history for this paper can be accessed here:http://www.biomedcentral.com/1472-6882/14/352/prepub

